# Predictive ability of severity scores and outcomes for mortality in kidney transplant recipients with coronavirus disease 2019 admitted to the intensive care unit: results from a Brazilian single-center cohort study

**DOI:** 10.1590/2175-8239-JBN-2021-0155

**Published:** 2022-02-09

**Authors:** Maria Bethânia Peruzzo, Lúcio Requião-Moura, Mônica Rica Nakamura, Laila Viana, Marina Cristelli, Hélio Tedesco-Silva, José Medina-Pestana

**Affiliations:** 1 Fundação Oswaldo Ramos, Hospital do Rim, Unidade de Terapia Intensiva, São Paulo, SP, Brasil.; 2 Fundação Oswaldo Ramos, Hospital do Rim, Departamento de Transplante, São Paulo, SP, Brasil.; 3 Universidade Federal de São Paulo, Departamento de Medicina, Disciplina de Nefrologia, São Paulo, SP, Brasil.

**Keywords:** Kidney Transplant Recipients, COVID-19, Intensive Care Units, Critical Illness, Injury Severity Score, Receptores de Transplante de Rim, COVID-19, Unidades de Terapia Intensiva, Estado Terminal, Escala de Gravidade do Ferimento

## Abstract

**Background:**

the predictive ability of severity scores for mortality in patients admitted to intensive care units is not well-known among kidney transplanted (KT) patients, especially those diagnosed with coronavirus disease 2019 (COVID-19). The purpose of the present study was to evaluate the predictive ability of severity scores for mortality in KT recipients.

**Methods:**

51 KT recipients with COVID-19 diagnosis were enrolled. The performance of the SOFA, SAPS 3, and APACHE IV tools in predicting mortality after COVID-19 was compared by the area under the ROC curve (AUC-ROC) and univariate Cox regression analysis was performed.

**Results:**

The 90-day cumulative incidence of death was 63.4%. Only APACHE IV score differed between survivors and nonsurvivors: 91.2±18.3 vs. 106.5±26.3, P = 0.03. The AUC- ROC of APACHE IV for predicting death was 0.706 (P = 0.04) and 0.656 (P = 0.06) at 7 and 90 days, respectively. Receiving a kidney from a deceased donor (HR = 3.16; P = 0.03), troponin levels at admission (HR for each ng/mL = 1.001; P = 0.03), APACHE IV score (HR for each 1 point = 1.02; P = 0.01), mechanical ventilation (MV) requirement (HR = 3.04; P = 0.002) and vasopressor use on the first day after ICU admission (HR = 3.85; P < 0.001) were associated with the 90-day mortality in the univariate analysis.

**Conclusion:**

KT recipients had high mortality, which was associated with type of donor, troponin levels, early use of vasopressors, and MV requirement. The other traditional severity scores investigated could not predict mortality.

## RESUMO

**Introdução:**

a capacidade preditiva dos escores de gravidade para mortalidade em pacientes admitidos em unidades de terapia intensiva não é bem conhecida entre pacientes transplantados renais (TR), especialmente aqueles diagnosticados com doença coronavírus 2019 (COVID-19). Este estudo avaliou a capacidade preditiva dos escores de gravidade para mortalidade em receptores de TR.

**Métodos:**

Foram inscritos 51 receptores de TR diagnosticados com COVID-19. O desempenho das ferramentas SOFA, SAPS 3, APACHE IV em predizer mortalidade após COVID-19 foi comparado pela área sob a curva ROC (AUC-ROC) e realizou-se análise de regressão univariada de Cox.

**Resultados:**

A incidência cumulativa de óbito em 90 dias foi 63,4%. Somente APACHE IV diferiu entre sobreviventes e não-sobreviventes: 91,2±18,3 vs. 106,5±26,3; P = 0,03. A AUC-ROC do APACHE IV para predizer óbito foi 0,706 (P = 0,04) e 0,656 (P = 0,06) aos 7 e 90 dias, respectivamente. Receber rim de doador falecido (HR = 3,16; P = 0,03), níveis de troponina na admissão (HR para cada ng/mL = 1,001; P = 0,03), escore APACHE IV (HR para cada 1 ponto = 1,02; P = 0,01), necessidade de ventilação mecânica (VM) (HR = 3,04; P = 0,002), uso de vasopressor no primeiro dia após admissão na UTI (HR = 3,85; P < 0,001) foram associados à mortalidade em 90 dias na análise univariada.

**Conclusão:**

Receptores de TR apresentaram alta mortalidade, associada ao tipo de doador, níveis de troponina, uso precoce de vasopressores e necessidade de VM. Os outros escores tradicionais de gravidade investigados não puderam predizer mortalidade.

## Introduction

By early January 2021, more than 90 million people worldwide had been diagnosed with COVID-19, and nearly 2 million had died^1^. The complex disease caused by the novel coronavirus severe acute respiratory syndrome coronavirus 2 (SARS-CoV-2) is characterized by a wide spectrum of clinical presentations, varying from asymptomatic infection to critical illness^2^. It is estimated that 50% of all infected patients have no symptoms or a very mild infection, which could go unnoticed^3^. Conversely, in some scenarios, patients can be quickly evaluated with a severe acute respiratory syndrome, with most requiring mechanical ventilation (MV), in addition to developing acute kidney injury (AKI), thrombosis, and cardiovascular events, resulting in a prolonged stay in intensive care units (ICUs) and a higher risk of mortality^4^. A worse clinical condition in coronavirus disease (COVID-19) patients has been correlated with several underlying conditions, such as older age, presence of comorbidities, and the net state of immunodepression^5,6^.

Of particular concern is immunodepression caused by the inevitable long-term exposure to immunosuppressive drugs in solid organ transplantation recipients^7^. Among them, kidney transplant (KT) recipients are more susceptible to severe forms of immunodepression conditions compared to other solid organ transplants recipients, considering the overload of immunodepression they are exposed to. They are also more prone to the presence of multiple comorbidities, such as diabetes, hypertension, obesity, and some degree of kidney function impairment^7,8^.

Among the many challenges due to COVID-19 in recent months, one has stimulated debates among ICU practitioners, namely finding the best scores to predict unfavorable outcomes. Traditional severity scales used in patients admitted in the ICU, such as the Sequential Organ Failure Assessment (SOFA)^9^, SAPS (Simplified Acute Physiology Score)^10^, and APACHE (Acute Physiology and Chronic Health Evaluation)^11^, have been updated and calibrated in the last decades^12^. However, it is unclear whether they are accurate for predicting outcomes and length of hospital stay in patients with COVID-19. Using these scores could be an additional challenge for specific populations, such as KT recipients^13^.

Therefore, the present pivotal study aimed to describe the main characteristics and death rate among the first KT recipients diagnosed with COVID-19 and treated in an ICU specialized in care of transplanted patients. Furthermore, the performance of three severity scoring systems (SOFA, SAPS, and APACHE), was analyzed to predict 90-day mortality.

## Methods

### Study design and population

This was a single-center, pivotal, retrospective, observational cohort study that enrolled a series of KT recipients diagnosed with COVID-19 and admitted to the ICU at the hospital specialized in KT recipients: Hospital do Rim, Brazil. This study was approved by the local Research Ethics Committee (approval number: CAAE 35321020.9.0000.8098). Patients or their relatives signed a consent form for inclusion.

KT recipients older than 18 years diagnosed with COVID-19 between March and August 2020 at the Hospital do Rim and admitted to the ICU were enrolled in the present study. In this period, 57 KT recipients were eligible, but one was excluded because the diagnostic test was not conclusive and five had a possible source of infection after ICU admission. The final follow-up date was December 1, 2020.

### COVID-19 diagnosis and supportive treatment

All enrolled patients received a COVID-19 diagnosis through a polymerase chain reaction test. The local approach for the clinical management of the SARS-CoV-2 infection included discontinuing immunosuppressive drugs, except steroids (prednisone), which were replaced with 0.5 mg/kg/day methylprednisolone, as well as 6.0 mg of dexamethasone after the results of the RECOVERY study^14^. The use of antibiotics was determined by the decision of physicians. No patients were enrolled in a clinical trial to investigate the clinical intervention for COVID-19.

### Data source, variables, and outcomes

The data were extracted from secondary and tertiary data sources in December 2020 and January 2021. The secondary data source was the electronic medical records, and the tertiary data source was a data frame built with data from all transplanted patients followed up in the local program and who were diagnosed with COVID-19.

We classified the variables into two main groups: demographic data and baseline characteristics at ICU admission. In the first group, we considered the following variables: age, sex, ethnicity, chronic kidney disease (CKD) etiology, time from transplant to ICU admission and time of transplantation (before 2018 or later), type of donor (deceased or living), immunosuppression schedule and recent acute rejection episode (defined as having an episode up to three months before the COVID-19 diagnosis), baseline estimated glomerular filtration rate (eGFR) estimated according to the CKD-EPI equation^15^, the Charlson comorbidity index^16^ at the time of COVID-19 diagnosis, and the reason for admission at the hospital.

In the second group, we considered the following variables at the time when patients were admitted to the ICU up to the first 24 h: reason for admission; antibiotic prescription; Glasgow Coma Scale score; ICU severity scores, such as SOFA, SAPS 3, and APACHE IV^9,10,17^; and some laboratory parameters, such as arterial blood gas analysis, levels of hemoglobin, leukocyte and lymphocyte, platelet, ferritin, troponin, and d-dimer; and eGFR according to the CKD-EPI equation. The SOFA score was considered in an additional time-point, the 24 hours SOFA assessed the following day after admission. Additionally, the 24-hour delta SOFA was evaluated by 24-hour SOFA - SOFA at admission. The clinical support analyzed on the first day after ICU admission was the use of vasopressors, MV, and renal replacement therapy (RRT) requirements. For AKI, we used the KDIGO definition and classification^18^.

The primary outcome was death, calculated as the 90-day cumulative incidence of mortality after ICU admission. Next, we analyzed the rate of mortality according to MV and RRT requirements. Finally, we compared the cumulative incidence of mortality according to the ICU severity score that presented the best performance in predicting the probability of death.

### Statistical analysis

Continuous variables were summarized as the mean and standard deviation if they had a normal distribution; otherwise, they were presented as the median and interquartile range (first and third IQR). Normality of data was assessed with the Shapiro-Wilk test. In two-tailed hypothesis testing, all variables were compared by head-to-head analysis, considering patients who died versus those who survived after ICU admission. Continuous variables were compared using Student’s *t*-test or the Mann-Whitney *U* test in accordance with the normality distribution, while categorical variables were compared using the *X^2^* or Fisher’s exact test, depending on the estimated frequency in a two-way table.

The cumulative incidence of mortality was estimated using the Kaplan-Meier method, and the comparison was performed using the log-rank test. Mortality rates according to intermediate outcomes, such as MV and RRT, were compared using *X^2^* test. To assess the baseline ICU severity scores that presented the best performance to predict the probability of mortality, we calculated the area under the curve (AUC) on receiver operating characteristic (ROC) curves based on mortality at 7, 28, and 90 days after ICU admission. Univariate analysis for risk of in-hospital death was performed by Cox regression for variables that presented a P-value < 0.10 (arbitrarily defined) in a head-to-head comparison excluding collinearity and the score that reached the best performance, and the probabilities are presented as hazard ratio (HR). The multiple imputation approach was used for missing linear values. Statistical analyses were conducted using SPSS version 26 (IBM Corp., Armonk, NY, USA), and statistical significance was defined as P < 0.05, with a 95% confidence interval (95%CI).

## Results

The demographic data are presented in Table 1. Patients were 51.0 ± 11.8 years old at ICU admission, 51% were male, and 51% was white. Patients scored 3.0 points in the Charlson comorbidity index, and the main cause of CKD was hypertension (37.3%), followed by diabetes (27.5%). Kidney transplantation had been carried out 47.9 months before the diagnosis of COVID-19, but 41.2% were transplanted in 2018 or earlier. Most patients had received a graft from a deceased donor (76.5%), and the most frequent immunosuppression schedule that patients were receiving was the combination of tacrolimus and mycophenolate (56.9%) followed by tacrolimus and azathioprine (19.7%). Other regimens are detailed in Table 1. Few patients (5.9%) had an acute rejection in the previous 3 months, and their baseline graft function was 32.9 mL/min/1.73 m^2^. The source of infection was community transmission in 45 patients (92.1%), in which the reason for hospital admission in 63.4% of patients (n = 19) was respiratory symptoms, followed by fever (12.2%, n = 5), gastrointestinal or cardiovascular symptoms (8.7% each, n = 4), and other symptoms (24.4%). The time between hospital admission and ICU admission was 2.0 (0.0; 17.0) days, and one-third of the patients were admitted directly to the ICU.

**Table 1:** Demographic Data

Variables	Total	End point		P	Missing
	N = 51	Non-survivors N = 32 (62.7)	Survivors N = 19 (37.3)		
Age (years)					0
*at the transplantation time*	*51.0 ± 11.8*	*52.4 ± 10.4*	*48.1 ± 14.0*	*0.31*	
*at the ICU admission time*	*58.6 ± 11.8*	*58.8 ± 10.7*	*54.7 ± 13.4*	0.23	
Sex (male) - n (%)	26 (51.0)	11 (46.9)	11 (57.9)	0.45	0
Ethnicity (white) - n (%)	26 (51.0)	15 (46.9)	11 (57.9)	0.45	0
CKD etiology - n (%)				0.23	0
*Diabetes*	*14 (27.5)*	*12 (37.5)*	*7 (36.8)*	*0.96*	
*Hypertension*	*19 (37.3)*	*11 (34.4)*	*3 (15.8)*		
*Polycystic disease*	*4 (9.8)*	*4 (12.5)*	*1 (5.3)*		
*Others/unknown*	*13 (30.0)*	*5 (15.6)*	*8 (42.1)*		
Time from transplant to ICU admission (months)	47.9 (8.7; 118.6)	36.7 (3.2; 137.1)	45.8 (10.5; 156.2)	0.73	
Era of transplantation (2018 or late)	21 (41.2)	12 (37.5)	9 (47.4)	0.49	0
Graft from deceased donor - n (%)	39 (76.5)	28 (87.5)	11 (57.9)	0.02	0
Immunosuppression - n (%)					0
*Induction (thymoglobulin)*	*47 (92.2)*	*32 (100)*	*15 (78.9)*	*0.02*	
*TAC-MPS*	*29 (56.9)*	*21 (65.6)*	*8 (42.1)*	*0.10*	
*TAC-AZA*	*9 (17.9)*	*6 (18.8)*	*3 (15.8)*	*0.55*	
*CSA-AZA*	*1 (9.8)*	*3 (9.4)*	*2 (10.5)*	*0.62*	
*TAC-iMTOR*	*4 (7.8)*	*1 (3.1)*	*3 (15.8)*	*0.14*	
*Other*	*8 (7.6)*	*1 (3.1)*	*3 (15.8)*	*0.14*	
Recent AR treatment - n (%)	3 (5.9)	2 (6.3)	1 (5.3)	0.69	0
Baseline eGFR (mL/min/1.73 m^2^)	32.9 (23.2; 62.3)	37.5 (12.9; 51.5)	53.1 (19.3; 83.4)	0.77	0
Charlson comorbidity index (n)	4.0 (2.0; 4.0)	4.0 (3.0; 4.7)	3.0 (2.0; 3.5)	0.23	0
Nosocomial transmission - n (%)	23 (45.1)	15 (46.9)	8 (42.1)	0.74	0

Legend: AR, acute rejection; AZA, azathioprine; CKD, chronic kidney disease; CSA, cyclosporin; eGFR, estimated glomerular filtration rate; ICU, intensive care unit; MPS, mycophenolate; mTORi, mammalian target of rapamycin inhibitors; TAC, tacrolimus.

Table 2 details the baseline characteristics at ICU admission. The main reason patients were admitted or transferred to the ICU was respiratory failure (72.5%), followed by neurologic impairment (11.8%). Of note, 90.2% were receiving antibiotics before ICU admission. Some laboratory parameters are summarized in Table 2. The eGFR was 24.5 mL/min/1.73 m^2^, which represented a negative slope of 6.5 (+0.2;-21.1) from baseline. Only one patient did not have AKI, while 5.9, 33.3, and 60.8% scored KDIGO 1, 2, and 3, respectively. The baseline ICU severity scores were: 54.0 in SAPS 3, 5.0 in SOFA, and 99.0 in APACHE IV (Table 3). After the first 24 h after ICU admission, 43.1% of patients required hemodynamic support with vasopressors, 39.2% required MV, and 43.2% required RRT.

**Table 2:** Baseline characteristic on admission to ICU

Variables	Total	End point		P	Missing
	N = 51	Non-survivor N = 32 (62.7)	Survivor N = 19 (37.3)		
Reason for ICU ad-mission - n (%)				0.05	0
*Respiratory failure*	*37 (72.5)*	*27 (84.4)*	*14 (73.7)*		
*Neurologic impairment*	*6 (11.8)*	*5 (15.6)*	*1 (5.3)*		
*Others*	*8 (15.7)*	-	*4 (21.0)*		
Antibiotic use before ICU admission - (n) %	46 (90.2)	31 (96.9)	15 (78.9)	0.06	0
Glasgow coma scale (index)	15.0 (15.0; 15.0)	15.0 (14.0; 15.0)	15.0 (15.0; 15.0)	0.05	0
PaO_2_/FiO_2_ (mmHg)	222 (164; 268)	204 (171; 351)	260 (161; 292)	0.61	2
PaO_2_ (mmHg)	70.0 (59.0; 87.5)	71.5 (65.2; 108.5)	65.0 (57.0; 79.0)	0.39	2
FiO_2_ (medians of %)	32.0 (25.0; 40.0)	35.0 (30.0; 40.0)	28.0 (23.0; 43.0)	0.02	1
PaCO_2_ (mmHg)	33.0 (27.7; 38.2)	23.5 (19.5; 29.0)	33.0 (28.5; 37.5)	0.14	1
Troponin (pg/mL)	33.4 (9.0; 84.6)	82.2 (45.9; 83.3)	8.7 (4.5; 23.2)	<0.0001	17
D-Dimer (ng/mL)	2.000 (1.090; 3.170)	4.450 (2.165; 11.272)	810 (370; 2.025)	0.005	24
Ferritin (ng/mL)	742 (492; 1.864)	995 (504; 1.518)	821 (557; 1.307)	0.92	21
Prothrombin activity (medians of %)	91.1 (79.9; 100.0)	88.1 (81.1; 97.2)	96.0 (82.0; 100.0)	0.71	1
eGFR (mL/min/1.73 m^2^)	24.5 (16.0; 43.5)	20.4 (9.4; 35.0)	43.5 (12.5; 57.5)	0.46	0
Hemoglobin (g/dL)	10.0 (8.9; 12.3)	9.0 (8.9; 9.4)	10.9 (9.7; 13.0)	0.95	0
Leucocytes (cells/mm^3^)	5.900 (4.500; 8.100)	5.300 (4.225; 12.075)	5.100 (4.000; 10.500)	0.90	0
Lymphocytes (cell/mm^3^)	558 (339; 908)	436 (351; 517)	363 (322; 1.123)	0.32	1
Platelets (cell/mm3 * 1000)	131 (100; 187)	127 (100; 215)	130 (111; 208)	0.43	0
Arterial pH (rate)	7.38 (7.29; 7.44)	7.42 (7.32; 7.48)	7.38 (7.26; 7.46)	0.57	0
Arterial bicarbonate (mEq/L)	20.6 ± 6.8 19.2 (15.0; 24.0)	19.5 ± 6.9 13.8 (11.8; 20.9)	22.4 ± 6.5 19.3 (14.0; 24.1)	0.15 0.04	0

Legend: ICU, intensive care unit; FiO_2_, fraction of inspired of oxygen; PaO_2_, arterial partial pressure of oxygen.

**Table 3:** Baseline ICU score indices and clinical support on the first day in ICU

Variables	Total	End point		P
	N = 51	Non-survivor N = 32 (62.7)	Survivor N = 19 (37.3)	
Baseline ICU severity scores				
SAPS 3	55.0 (50.0; 61.0)	55.5 (53.2; 63.0)	47.0 (45.0; 50.0)	0.13
SOFA (at admission)	5.0 (4.0; 8.0)	4.0 (2.5; 10.0)	4.0 (4.0; 4.0; 6.5)	0.29
24-hour SOFA	6.0 (3.0; 11)	6.0 (3.0; 10.5)	6.0 (3.0; 10.5)	0.94
24-hour Delta SOFA	0.0 (-4.0; 4.0)	-1.0 (-4.5; 3.0)	0.0 (-2.0; 7.0)	0.19
APACHE IV	100.8 ± 24.6	106.5 ± 26.3	91.2 ± 18.3	0.03
Clinical support on the first day - n (%)				
Use of vasopressors	22 (43.1)	20 (62.5)	2 (10.5)	< 0.001
Mechanical ventilation	20 (39.2)	18 (56.3)	2 (10.5)	0.001
Renal replacement therapy	22 (43.2)	16 (50.0)	6 (31.6)	0.20

Legend: APACHE, Acute Physiology and Chronic Health Evaluation; ICU: intensive care unit; SAPS, Simplified Acute Physiology Score; SOFA Sequential Organ Failure Assessment.

**Figure 1:**
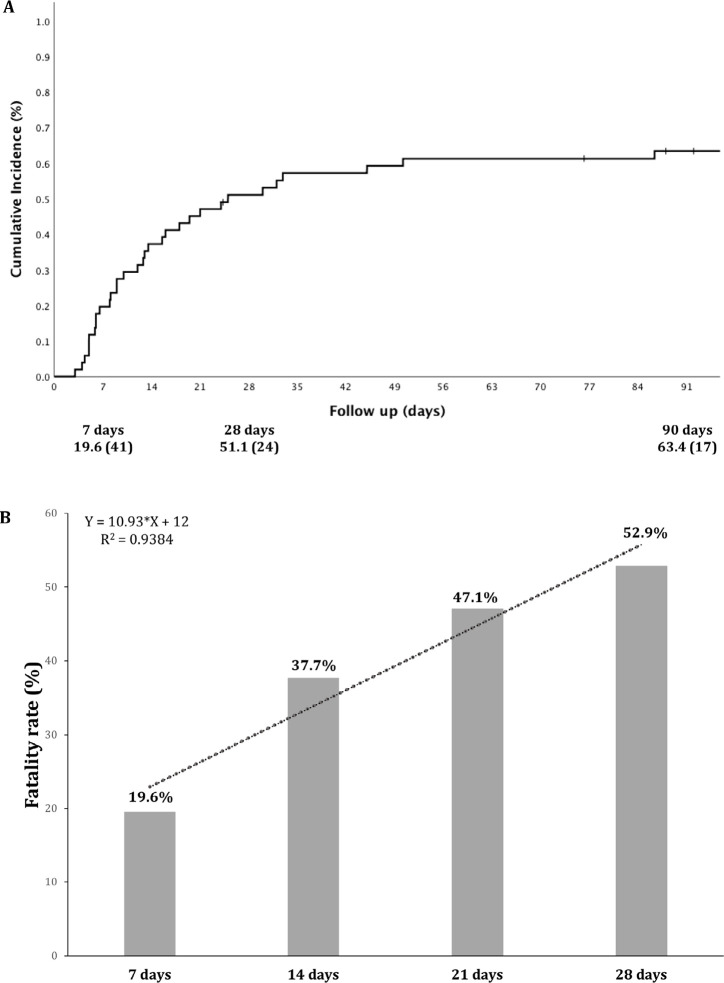
Cumulative incidence of death in critically ill kidney transplant recipients diagnosed with COVID-10. 1A: 90-day cumulative incidence of death; 1B: Cumulative rate of fatality from the 7th to the 28th day after ICU admission.

After ICU admission, the 90-day cumulative incidence of death was 63.4% (Figure 1A), and the cumulative rate had a linear trend line of increment from the 7th day (19.6%) to the 28th day (52.9%) after ICU admission, as shown in Figure 1B: Y = 10.93 * X + 12; R^2^ = 0.94. In Figure 1A, a decrease in the number of new deaths after 28 days is shown, with an incremental incidence of 9.3% from the 28th to the 90th day. The leading cause of death was septic shock due to secondary infection (17; 53.1%), followed by respiratory failure (8; 25.0%), cardiovascular events (4; 12.5%), and other causes (2; 9.4%).

The head-to-head analysis showed that recipients who had received a graft from a deceased donor (87.5% vs. 57.9%, P = 0.02) died more frequently than those who received a graft from a living donor. ICU admission due to respiratory failure (83.3 vs. 70.6%) and neurologic impairment (16.7% vs. 5.9%) was more frequent in patients who died (P = 0.04). The results of all variables analyzed in the head-to-head analysis are detailed in Tables 1 and 2.

We compared the baseline ICU severity scores and the clinical support on the first day after admission among patients who died and those who survived (Table 3). We did not find a difference between SOFA, 24-hour SOFA, and SAPS 3 scores and the 24-hour delta SOFA; however, the APACHE IV score was higher in patients who died: 106.5 ± 26.3 vs. 91.2±18.3, P = 0.03. As expected, vasopressors and MV requirement on the first day after ICU admission were more frequent in patients who died than in survivors: 62.5 vs. 10.5%, P < 0.001, and 56.3% vs. 10.5%, P = 0.001, respectively. The requirement for RRT was not different. Among all patients who required MV, the fatality rate was 90%, while in patients who did not, it was 45.2% (P = 0.001). Furthermore, the fatality rate was numerically higher among patients who required RRT (72.7% vs. 55.2%); however, this difference was not significant (P = 0.20). Among the survivors, eGFR 3 months after ICU admission was 27.5 (13.8; 49.1) mL/min/1.73 m^2^. Seven patients who required RRT survived and four partially recovered the graft function, with eGFR ranging from 11.7 to 43.2 mL/min/1.73 m^2^.

**Figure 2:**
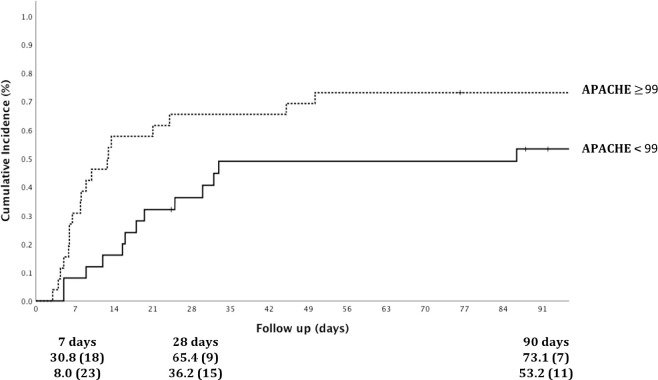
Ninety-day cumulative incidence of death according to APACHE IV. The APACHE IV was dichotomized in the median value of 99.

We analyzed the performance of ICU severity scores in predicting the probability of death at 7, 28, and 90 days after ICU admission. These results are detailed in Table 4. Among them, the scoring system with the best predictive ability for mortality at each time point was APACHE IV. The AUC in the ROC curve after 7 days was 0.706 (95% CI = 0.539; 0.873, P = 0.04), whereas it was 0.656 (95% CI = 0.504; 0.808, P = 0.06) in predicting 90-day mortality. Figure 2 shows the 90-day cumulative mortality dichotomized by the APACHE IV median value. Patients who had an APACHE IV score ≥ 99 presented a 90-day cumulative incidence of 73.1% versus 53.2% in those who presented a lower score (P = 0.04).

**Table 4:** Receiver operating characteristic curve for mortality at three time-points according to the baseline ICU severity scores

Score	AUC	95% CI		P
		Lower	Upper	
ROC curve for death in 7 days				
SAPS 3	0.535	0.327	0.744	0.73
SOFA	0.546	0.326	0.767	0.65
APACHE IV	0.706	0.539	0.873	0.04
ROC curve for death in 28 days				
SAPS 3	0.634	0.474	0.794	0.10
SOFA	0.528	0.367	0.689	0.73
APACHE IV	0.670	0.520	0.819	0.04
ROC curve for death in 90 days				
SAPS 3	0.628	0.461	0.795	0.13
SOFA	0.588	0.432	0.744	0.30
APACHE IV	0.656	0.504	0.808	0.06

Legend: APACHE, Acute Physiology and Chronic Health Evaluation; ROC, Receiver operating characteristic; SAPS, Simplified Acute Physiology Score; SOFA Sequential Organ Failure Assessment.

In the univariate analysis (Table 5), the risk of death was higher in patients who had received a graft from a deceased donor (HR = 3.16, 95% CI= 1.10-9.05; P = 0.03), in those who had higher troponin levels at admission (HR = 1.001; 95% CI = 1.000-1.002; P = 0.03), higher APACHE IV score (HR = 1.02; 95%CI = 1.004-1.03; P = 0.01), and those who required MV (HR = 3.04; 95CI = 1.48-6.24; P = 0.002) and vasopressors (HR = 3.85; 95%CI = 1.84-8.06; P < 0.001) on the first day after ICU admission.

**Table 5:** Univariate analysis for risk of in-hospital death

Variables	Univariate	
	HR (95% CI)	P
APACHE IV (for each 1 point)	1.02 (1.004-1.03)	0.01
Troponin at admission (for each 1 pg/mL)	1.001 (1.000-1.002)	0.03
Mechanical ventilation in the first day after ICU admission (yes vs. no)	3.04 (1.48-6.24)	0.002
Deceased donor (yes vs. no)	3.16 (1.10-9.05)	0.03
Use of vasopressors in the first day after ICU admission (yes vs. no)	3.85 (1.84-8.06)	< 0.001

Footnotes: Variables excluded and why: use of thymoglobulin as immunosuppressive induction due to collinearity with the type of donor; different reasons for ICU admission, mechanical ventilation, and Glasgow Coma Scale score was excluded because of collinearity with APACHE. The use of tacrolimus associated with mycophenolate as maintenance immunosuppression, D-dimer at admission, and use of antibiotics before ICU admission reached a P-value > 0.05. Legend: CI, confidence interval; ICU, intensive care unit; HR, hazard ratio; TAC-MPS, tacrolimus associated with mycophenolate.

## Discussion

In the present study, the outcomes of KT recipients diagnosed with COVID-19 and required ICU admission for the clinical management of infections in the first six months of the pandemic were analyzed. Additionally, we explored the performance of three traditional scores of severity in predicting the probability of death: SOFA, SAPS, and APACHE.

It has been estimated that 30% of all KT recipients diagnosed with COVID-19 (19) need ICU care. Besides, current data for this population indicates higher rates of intermediate outcomes than non-transplanted patients, such as MV and AKI requiring RRT^8,19,20^. In the present study, 57% required MV, 40% of that in the first 24 h after ICU admission. Surprisingly, it was not different from occurs in the general population. In a prospective multicenter cohort study that included more than 4,000 patients in Europe, the consortium COVID-ICU Investigators found that 63% of patients were intubated during the first 24 h after ICU admission^21^. There are some possible explanations for this early incidence of intubation. As expected, the main cause of ICU admission was respiratory failure due to the severe acute respiratory syndrome, similar to our patients. In the first months of the pandemic, patients were admitted in a very severe condition^21^. Furthermore, there was a clear recommendation for early intubation at the beginning of the pandemic, owing to the risk of exposure of healthcare workers^22^, which was modified over time after noninvasive strategies were shown to be safe and efficient^23^.

Another study found that disease severity in these patients was due to the high rate of early vasopressor requirement: 43% in the first 24 h. Although early ventilation was the best protective strategy^24^, some patients needed deep sedation for controlled pressure and early prone position, which is associated with some degree of hemodynamic instability^25^. Thereafter, ICU staff administered fluids cautiously to avoid volume overload in patients with severe respiratory impairment, considering the high incidence of myocardial dysfunction associated with COVID-19^26,27^. Of note, 13% of patients who needed vasopressors on the first day had septic shock, which has been the main cause of death. Taken together, these features increased the risk to 3.78 when patients required early vasopressor treatment. In another study that evaluated KT recipients with COVID-19, septic shock was reported as the main cause of death; however, it is important to consider that sepsis due to a secondary infection or opportunist infections lead to longer ICU stays^8^. Finally, the rate of early RRT requirement was very high because baseline graft function was very low, decreasing by more than 20% before ICU admission and leading to an AKI incidence of 98%; most cases were scored as KDIGO 3. It is well known that the risk of AKI is significantly associated with age, diabetes, previous cardiovascular disease, and MV or vasopressor requirement^6^, which are highly prevalent in our study population.

Herein, we observed 62.7% fatality among patients admitted to the ICU and 90.0% among those who required MV. We decided to extend the follow-up to 90 days rather than 28 because initial evidence has demonstrated a significant number of patients in the ICU after a short time^14,26,28^. Figure 1B shows the linear tendency of increasing death rate from the 7th to 28th day; however, the additional cumulative mortality after 28 days was nearly 10% (Figure 1.A). In the first reports from China, the fatality rate among critically ill patients ranged from 50 to 62%^26,28^, which were not higher than those observed among the first 250,0000 Brazilians with COVID-19 who received in-hospital clinical management between February and August 2020^29^. It is important to highlight that in-hospital patients from the general population were 60 years old on average, as observed in our patients (58.6 years old). Similarly, in-hospital fatality in Brazilian patients was 38%, reaching 59% among those admitted to the ICU, and 80% when MV was required.

Since the first patients infected by the novel coronavirus were identified, a great variety of clinical presentations have been reported. However, the potential for progression to severe acute respiratory syndrome has been of great concern worldwide^2,4,30^. Indeed, many affected patients have no symptoms or present with discrete or mild flu syndrome; therefore, one of the great challenges in the first months of the pandemic was to find the most accurate prognostic markers, and the first reported underlying conditions were older age and presence of comorbidities, such as hypertension, diabetes, chronic cardiovascular or pulmonary diseases, and some degree of kidney function impairment^5,6^. In this scenario, KT recipients are considered to have a higher susceptibility to infection and worse outcomes due to the chronic and unavoidable use of immunosuppressive drugs and the presence of comorbidities^8,19,20^.

Most variables that have a defined risk among patients with COVID-19, including KT recipients, have the most useful severity scores in predicting early or in-hospital mortality and length of hospital stay in critically ill patients, such as SOFA, SAPS, and APACHE. Although these scores had been updated and calibrated in the last decades, it was not clear whether they were accurate enough in COVID-19 patients. For instance, the first studies that included patients with COVID-19 found some nontraditional markers as prognostic factors, such as ferritin, d-dimer, lactate dehydrogenase, and lymphopenia^4,6,30^. Furthermore, when critically ill COVID-19 patients admitted to the ICU were compared with the historical group admitted to the same unit in the same months of the year before the pandemic, SAPS 3 and SOFA scores were lower in patients with COVID-19^31^. Conversely, in a study conducted by European COVID-ICU investigators, SOFA was independently related to 90-day mortality; even though the disease primarily affects the lungs, the kidney and cardiovascular components of SOFA were the most strongly associated to unfavorable outcomes^21^. It is interesting to note that, in that study, immunosuppression was one of the variables independently associated to death. Moreover, in a Chinese study that enrolled 140 critically ill COVID-19 patients, three or more points in the SOFA score presented 90% sensitivity and 97% negative predictive value for mortality^32^. Finally, in another Chinese cohort that included 154 patients, APACHE II was independently associated with hospital mortality: each point significantly increased the risk by 7%^33^. These initial findings suggest that, although COVID-19 is an incomparable systemic disease caused by a respiratory virus, some traditional scores should be used to predict outcomes in critically ill patients; however, further calibration would be desirable.

To the best of our knowledge, this is the first study to explore the performance of traditional severity indices in predicting mortality in critically ill KT recipients diagnosed with COVID-19. Previously, our group had evaluated the performance of SOFA in predicting in-hospital mortality among patients admitted to the same ICU due to septic shock, wherein each point in 24 h delta SOFA increased the risk of death by 70%^13^, but to date no score has been calibrated for KT recipients. Herein, APACHE IV achieved the best outcome in predicting 7- and 28-day mortality, with a trend for 90-day mortality. Furthermore, patients who presented with APACHE IV > 99 had a higher and significantly cumulative mortality.

Our study has several limitations, including the small number of patients, its retrospective and pivotal nature, and a large number of missing values of some important variables, such as troponin, d-dimer, and ferritin levels, which had better outcomes in predicting risk of death in COVID-19 patients in previous studies. Considering these limitations, it is not possible to conclude that these scoring systmes are inadequate for KT recipients. Furthermore, due to the small number of patients and survivors and the low rate of events per variable, the potential predictors of death could not be evaluated in multivariate modeling, being the results limited to a univariate analysis. However, our results provide preliminary information on a relevant field in the context of the COVID-19 pandemic, and future investigations must be conducted to explore some of the questions highlighted in the present study. This is the first Brazilian study that examined this specific population admitted to the ICU and its main outcomes. It is important to emphasize that Brazil has the largest public transplant program and performs the second largest number of kidney transplants in the world, and the COVID-19 pandemic is having a significant impact on this population. Further information is welcome to improve the clinical management of KT recipients, mainly those that evolve to critically ill conditions.

## Conclusion

Although this is a small pivotal cohort study, it highlights a relevant field of investigation: the prediction of unfavorable outcomes in KT recipients with COVID-19. As expected, the 90-day mortality among KT recipients was very high and independently associated with the use of vasopressors on the first day after ICU admission. Receiving a kidney from a deceased donor was also related to mortality, despite the long period between transplantation and SARS-CoV-2 infection. Finally, we observed that, among SOFA, SAPS3, and APACHE IV, the latter exhibited the best outcomes in predicting early and 90-day mortality.

## Abbreviations

AKI: acute kidney injury
APACHE: Acute Physiology and Chronic Health Evaluation
AR: acute rejection
AZA: azathioprine
CKD: chronic kidney disease
COVID-19: coronavirus disease
CSA: cyclosporin
eGFR: estimated glomerular filtration rate
FiO_2_: fraction of inspired oxygen
ICU: intensive care unit
KT: kidney transplant
MPS: mycophenolate
mTORi: mammalian target of rapamycin inhibitors
MV: mechanical ventilation
PaO_2_: arterial partial pressure of oxygen
RRT: renal replacement therapy
SAPS: Simplified Acute Physiology Score
SARS-CoV-2: severe acute respiratory syndrome coronavirus 2
SOFA: Sequential Organ Failure Assessment
TAC: tacrolimus
